# Expression Pattern of Thyroid Hormone Transporters in the Postnatal Mouse Brain

**DOI:** 10.3389/fendo.2014.00092

**Published:** 2014-06-18

**Authors:** Julia Müller, Heike Heuer

**Affiliations:** ^1^Leibniz Institute for Age Research – Fritz Lipmann Institute, Jena, Germany; ^2^Leibniz Research Institute for Environmental Medicine, Düsseldorf, Germany

**Keywords:** Mct8, Mct10, Oatp1c1, Lat1, Lat2, T3, T4, CNS

## Abstract

For a comprehensive description of the tissue-specific thyroidal state under normal as well as under pathophysiological conditions it is of utmost importance to include thyroid hormone (TH) transporters in the analysis as well. The current knowledge of the cell-specific repertoire of TH transporters, however, is still rather limited, although several TH transporting proteins have been identified. Here, we describe the temporal and spatial distribution pattern of the most prominent TH transporters in the postnatal mouse brain. For that purpose, we performed radioactive *in situ* hybridization studies in order to analyze the cellular mRNA expression pattern of the monocarboxylate transporters Mct8 and Mct10, the L-type amino acid transporters Lat1 and Lat2 as well as the organic anion transporting peptide Oatp1c1 at different postnatal time points. Highest TH transporter expression levels in the CNS were observed at postnatal day 6 and 12, while hybridization signal intensities visibly declined after the second postnatal week. The only exception was Mct10 for which the strongest signals could be observed in white matter regions at postnatal day 21 indicating that this transporter is preferentially expressed in mature oligodendrocytes. Whereas Mct8 and Lat2 showed an overlapping neuronal mRNA expression pattern in the cerebral cortex, hippocampus, and in the hypothalamus, Oatp1c1 and Lat1 specific signals were most prominent in capillary endothelial cells throughout the CNS. In the choroid plexus, expression of three transporters (Mct8, Lat2, and Oatp1c1) could be detected, whereas in other brain areas (e.g., striatum, thalamus, and brain stem nuclei) only one of the transporter candidates appeared to be present. Overall, our study revealed a distinct mRNA distribution pattern for each of the TH transporter candidates. Further studies will reveal to which extent these transporters contribute to the cell-specific TH uptake and efflux in the mouse CNS.

## Introduction

Thyroid hormone (TH) action requires the presence of TH transporters that facilitate its cellular uptake and efflux (1–4). Since TH metabolizing deiodinases as well as TH receptors are intracellularly active, TH transporter deficiency can greatly compromise tissue TH homeostasis. As the most prominent example, inactivating mutations in the monocarboxylate transporter 8 (MCT8; SCL16A2) gene, which encodes a very specific TH transporter (5), result in an abnormal serum TH profile with highly elevated levels of the receptor active hormone T3 (3,3^′^,5,-triiodothyronine) and low T4 concentrations of the prohormone T4 (3,3^′^,5,5^′^-tetraiodothyronine) (6–8). Moreover, patients with MCT8 mutations suffer from a severe form of psychomotor retardation suggesting that in the absence of MCT8, neural differentiation and function is severely impaired possibly due to insufficient TH supply during critical stages of development (9). The exact role of MCT8 is still enigmatic since only limited information is currently available concerning the cellular localization of MCT8 in the developing and adult human CNS (10–12).

Studies of Mct8 knockout (ko) mice unequivocally revealed that Mct8 is not the only protein involved in TH transport processes in the murine CNS (11, 13, 14). Although immunohistochemical studies and *in situ* hybridization (ISH) experiments showed pronounced Mct8 expression in distinct neuronal populations of the cerebral cortex, hippocampus, striatum, hypothalamus, and cerebellum (15), Mct8 ko mice do not display overt neurological symptoms (11), although they faithfully replicate the abnormal serum TH parameters characteristic for human MCT8 deficiency. These Mct8 ko mice also do not show immunohistochemically any abnormalities such as a delayed Purkinje cell development or an altered differentiation of inhibitory neurons in the cerebral cortex, both strong neuronal indicators for TH deprivation (11, 14). Based on these observations, it was hypothesized that other TH transporting proteins can compensate for the absence of Mct8 in the mouse CNS. Indeed, analysis of primary neuronal cultures from the mouse cortex revealed a collaborative action of Mct8 and the L-type amino acid transporter Lat2 that in addition to large neutral amino acids also accepts TH as substrates (11). Which transporters, however, are co-expressed with Mct8 *in vivo* has not been sufficiently addressed yet.

Apart from neurons, Mct8 is present in capillary endothelial cells as well as in the choroid plexus structures, thus in cells that build up the blood–brain (BBB) and the blood–cerebrospinal fluid-barrier (BCSFB), respectively (10, 11, 15). That Mct8 is indeed involved in the passage of TH via the BBB and/or BCSFB could be demonstrated by *in vivo* transport studies. Uptake of T3 from the circulation into the CNS was strongly diminished in Mct8 ko mice, whereas the transport of T4 was only mildly compromised (14, 16) due to the presence of the T4 transporting organic anion transporting peptide Oatp1c1 (Slco1c1) (17–19). Indeed, the generation and analysis of Mct8/Oatp1c1 double knockout (dko) mice confirmed the physiological significance of both TH transporters for proper TH homeostasis in the murine brain since the brain T3 and T4 content of these animals was reduced to 10% of wild-type levels (20). Obviously, Mct8 and Oatp1c1 act as a pair in mediating TH access to the CNS, although the residual TH amounts found in brain homogenates of Mct8/Oatp1c1 dko mice suggest that additional, not yet identified TH transporters contribute to this process as well.

From all these mouse studies it became strikingly clear that a detailed determination of the tissue- and cell-specific repertoire of TH transporters is highly needed for a comprehensive understanding of TH trafficking, metabolism, and action under normal as well as pathological conditions. Recently, Braun et al. generated a developmental profile of TH transporter expression patterns in different brain regions by performing western blot analysis and qPCR studies (21). However, these studies did not provide any information regarding the cellular localization pattern. We therefore conducted a series of ISH experiments that allow the temporal and spatial analysis of TH transporter expression with a cellular resolution in the postnatal mouse brain. In addition to Mct8, Oatp1c1, and Lat2, we included the aromatic amino acid transporter Mct10 (Slc16a10) and the L-type amino acid transporter Lat1 (Slc7a5) in our study since both proteins have been shown to accept TH as substrates (22, 23) and may play an important role for TH transmembrane passage in the CNS as well.

## Materials and Methods

### Animals

All mice were provided with standard laboratory chow and tap water *ad libitum* and were kept at constant temperature (22°C) and controlled light cycle (12 h light, 12 h dark). Male wild-type mice were killed in accordance with local regulations (TLLV Thüringen, Erfurt, Germany; approval number TöA-FLI149-08) by CO_2_ at different postnatal time points (P6, P12, P21, P84) and brains were frozen in isopentane cooled on dry ice. For each time point, three brains were prepared. Coronal cryosections with a thickness of 20 μm were cut with a cryostat and thaw mounted on superfrost slides (Thermo Scientific). Slides were stored at −80°C until further processing.

### *In situ* hybridization histochemistry

A cDNA fragment corresponding to nt 1251–1876 of mouse Mct8 (GenBank accession number AF045692), nt 911–1663 of mouse Mct10 (NM_001114332), nt 921–1357 of mouse Lat1 (NM_011404.3), nt 972–1457 of mouse Lat2 (NM-016972.2), nt 360–470 of mouse Oatp1c1 (NM_021471.1) were generated by PCR and subcloned into the pGEM-T Easy Vector (Promega). Radiolabeled riboprobes were generated by *in vitro* transcription using ^35^S-UTP as labeled substrate (Hartmann Analytik, Braunschweig, Germany). ISH was carried out as published elsewhere (24). In brief, frozen sections were air-dried, followed by an 1-h fixation in a 4% phosphate-buffered paraformaldehyde (PFA) solution (pH 7.4) and then permeabilized by incubation in 0.4% Triton-X 100/PBS for 10 min. Acetylation was carried out in 0.1 M triethanolamine (pH 8.0) containing 0.25% (v/v) acetic anhydride. Sections were dehydrated and then covered with hybridization mix containing cRNA probes diluted in hybridization buffer (50% formamide, 10% dextran sulfate, 0.6 M NaCl, 10 mM Tris–HCl pH 7.5, 1× Denhardt’s solution, 100 μg/ml sonicated salmon sperm DNA, 1 mM EDTA, and 0.5 mg/ml t-RNA). 35S-labeled riboprobes were diluted in hybridization buffer to a final concentration of 1 × 10^4^ cpm/μl (Mct8) or 2 × 10^4^ cpm/μl (Lat1, Lat2, Oatp1c1). Hybridization was performed over night at 58°C. Slides were rinsed in 2× standard saline citrate (0.3 M NaCl and 0.03 M sodium citrate, pH 7.0) and subsequently treated with ribonuclease A/T1 at 37°C for 30 min. Final washes were carried out in 0.2× standard saline citrate at 65°C for 1 h. For detecting radioactive hybridization signals, the sections were dehydrated and then exposed to x-ray film (BioMax MR, Eastman Kodak Co.) for 24–48 h. Thereafter, sections were dipped in Kodak NTB nuclear emulsion (Kodak) and stored at 4°C for 8 days (Mct8), 7 days (Lat1), 6 days (Lat2), or 11 days (Oatp1c1). Autoradiograms were developed and analyzed under dark-field illumination. As controls, consecutive brain sections were probed with the sense-strand probes of the same size and specific activity and processed in the same manner as the sections covered with the respective antisense probe. For none of these sense probes has a positive signal been encountered.

## Results

**Figure 1:**
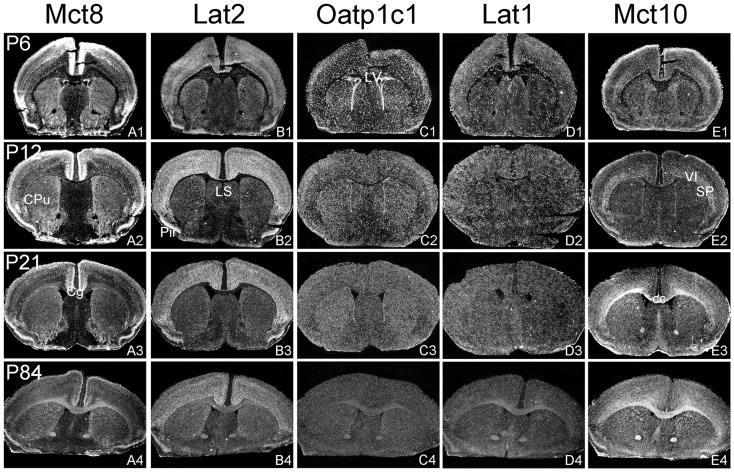
**Illustration of TH transporter mRNA expression in the murine forebrain**. Coronal cryosections of mouse brains collected at different postnatal days were hybridized with radioactively labeled riboprobes specific for Mct8 **(A1–A4)**, Lat2 **(B1–B4)**, Oatp1c1 **(C1–C4)**, Lat1 **(D1–D4)**, and Mct10 **(E1–E4)**. Dark-field autoradiograms illustrate the mRNA expression patterns at the level of the striatum (CPu, caudate–putamen) around Bregma 0.5 mm. VI, layer 6 of the cerebral cortex; cc, corpus callosum; Cg, cingulate cortex, LV, lateral ventricle; Pir, piriform cortex; SP, subplate neurons (cerebral cortex).

**Figure 2:**
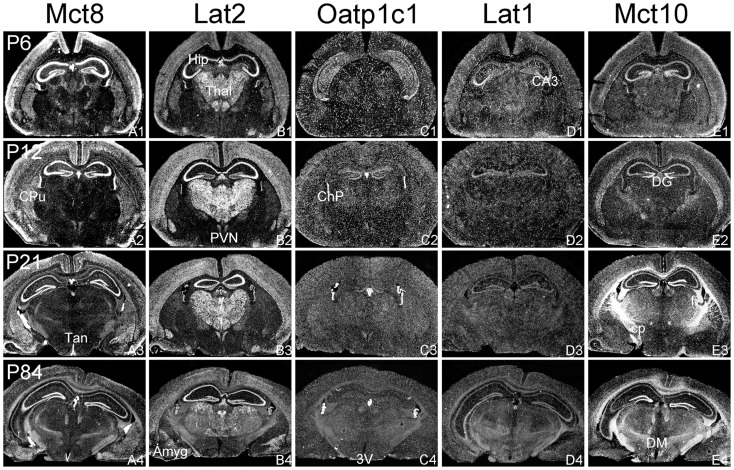
**mRNA distribution patterns of TH transporters in the murine hippocampus and diencephalon**. Mct8 **(A1–A4)** and Lat2 **(B1–B4)** exhibited an overlapping mRNA expression in the cerebral cortex, hippocampus (Hip), amygdala (Amyg), hypothalamic paraventricular nucleus (PVN), and choroid plexus (ChP). Mct8 but not Lat2 is highly expressed in tanycytes (Tan) lining the third ventricle (3V). In contrast, Lat2 but not Mct8 is strongly expressed in thalamic nuclei (Thal). Elongated hybridization signals scattered throughout the CNS indicate a capillary localization of Oatp1c1 **(C1–C4)** and Lat1 **(D1–D4)**. In addition, Lat1 is present in the hippocampal CA3 neurons at postnatal day P6. Mct10 **(E1–E4)** is also temporarily expressed in hippocampal and cortical neurons. At P21, however, highest hybridization signal intensities are observed in white matter areas [e.g., in the cerebral peduncle (cp) or the hippocampal fimbria (fi)]. DG, dentate gyrus; DM, dorsomedial hypothalamic nucleus.

**Figure 3:**
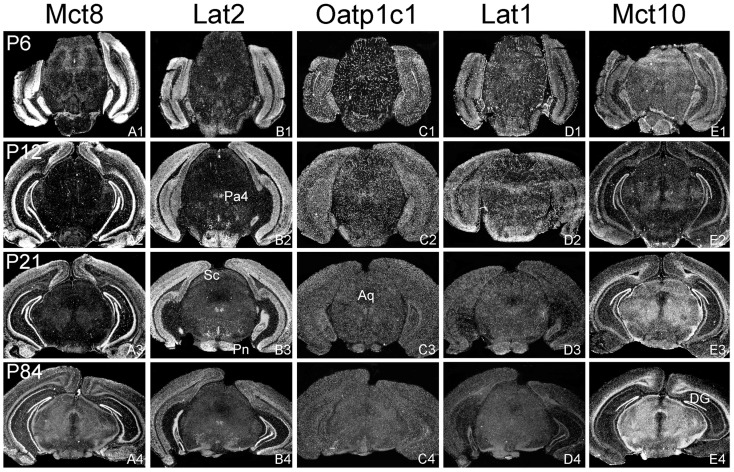
**Differential expression of TH transporters in midbrain regions**. Whereas Mct8 mRNA expression is close to the detection limit in the midbrain area **(A1–A4)**, Lat2 specific hybridization signals **(B1–B4)** are observed in distinct nuclei such as the paratrochlear nucleus (Pa4) and the pontine nuclei (Pn). Weak signals for Lat2 are also found in the superior colliculi (Sc). Oatp1c1 **(C1–C4)** and Lat1 **(D1–D5)** exhibit a capillary expression pattern whereas Mct10 **(E1–E4)** is strongly expressed in the white matter and dentate gyrus neurons (DG). Aq, aqueduct.

**Figure 4:**
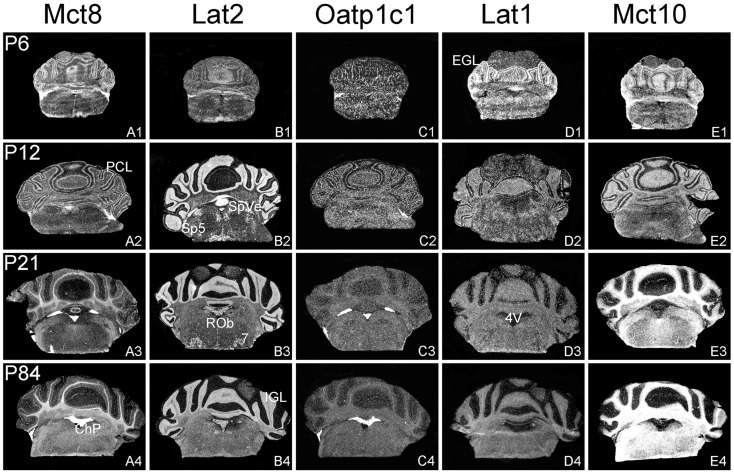
**Cerebellar and brainstem expression pattern of TH transporters**. In the developing cerebellum, Mct8 specific hybridization signals can be found in the external granule cell layer (EGL) and the Purkinje cell layer (PCL) **(A1,A2)** whereas after weaning Mct8 mRNA expression is largely restricted to choroid plexus structures of the fourth ventricle (4V) **(A3,A4)**. In comparison, Lat2 mRNA levels are high in the internal granule cell layer (IGL) indicating that mature granule cells express this transporter **(B1–B4)**. Lat2 specific signals could also be found in various brainstem nuclei such as the facial nucleus (7), the raphe obscurus nucleus (ROb), the spinal 5 nucleus (Sp5), and the spinal vestibular nucleus (SpVe). Oatp1c1 is highly expressed in choroid plexus as well as in endothelial cells **(C1–C4)** where also Lat1 staining can be observed. In addition, Lat1 expression is found in the EGL and PCL of the cerebellar cortex at P6 **(D1–D4)**. Mct10 expression pattern indicates a pronounced localization in mature oligodendrocytes **(E1–E4)**.

The aim of the current study was to perform a comparative analysis of TH transporter expression patterns in the murine CNS at different postnatal time point. In particular, we aimed to determine the cellular distribution pattern of the transporters Mct8 (Slc16a2), Mct10 (Slc16a10), Lat1 (Slc7a5), Lat2 (Slc7a8), and Oatp1c1 (Slco1c1) that have all been described to facilitate the cellular transport of TH (25). Since only for a subset of these proteins specific antibodies are available, we employed a highly sensitive ISH technique using ^35^S-labeled RNA probes. Brains were collected from C57/Bl6 male mice at postnatal day 6 (P6), P12, P21, and P84 and consecutive frozen brain sections were subjected to the ISH procedure as described previously (24). For the description of the mRNA distribution at a cellular resolution, nuclear emulsion coated slides were examined under dark-field illumination using a light-microscope. Each antisense probe indeed produced a positive hybridization signal with a highly distinct cellular distribution pattern as visualized exemplarily in Figures 1–4 for four different anatomical regions. In particular, Figure 1 illustrates cortical and striatal expression at around Bregma 0.50 mm; Figure 2 (between Bregma −1.8 and −2.2 mm) shows mRNA expression patterns in the cortex, hippocampus, thalamus, and hypothalamus; Figure 3 depicts TH transporter expression between Bregma −3.4 and −4.4 including mesencephalic areas; Figure 4 shows cerebellar and brainstem expression pattern around Bregma −6.2 [according to Franklin and Paxinos (26)].

### Distribution pattern of Mct8 and Lat2

In agreement with our previous analysis (15) and immunohistochemical studies (11), Mct8 was found in different brain areas with strongest expression levels in pyramidal and granule cells of the hippocampus, in the choroid plexus and in tanycytes of the third ventricle (Figures 2A1–A4). Pronounced hybridization signals were also found in the upper layers of the cerebral cortex, throughout the striatum (Figures 1A1–A4) as well as in cerebellar Purkinje cells particularly during the first postnatal weeks (Figures 4A1–A4). In these areas, Mct8 signal intensities were visibly reduced in the adult animals indicating a temporal decline in expression. A similar trend was also observed for Lat2 that exhibited an overlapping expression pattern with Mct8 in distinct areas such as the choroid plexus, the hippocampus, the cerebral cortex, and the hypothalamic neurons of the PVN (Figures 2B1–B4). Highest mRNA expression of Lat2, however, was observed throughout the thalamic region, an area devoid of Mct8 specific hybridization signals (Figures 2B1–B4). Lat2 specific mRNA signals were also found in the pontine nucleus (Figures 3B1–B4), various brain stem nuclei such as the facial nucleus, hypoglossal nucleus, and the raphe nuclei (Figures 4B1–B4) where Mct8 expression could not be detected. Another example for a complementary expression of Mct8 and Lat2 was observed in the cerebellar cortex where Mct8 transcript levels could be detected in granule precursor cells of the external granule cell layer at P6 as well as in developing Purkinje cells (at P6 and P12), whereas Lat2 was most intensely expressed in mature granule cells of the internal granule cell layer (Figures 4A1–A4,B1–B4). Overall, our results indicate a co-expression of both TH transporters only in distinct areas of the mouse CNS.

### Distribution pattern of Oatp1c1 and Lat1

Our ISH analysis confirmed published studies indicating a preferential expression of Oatp1c1 in choroid plexus structures, ventricle ependymal cells, and capillary endothelial cells (Figures 1C1–C4 and 2C1–C4) (10, 18, 27). In addition to the elongated hybridization signals typically for vessel structures, ISH analysis for Oatp1c1 also revealed scattered signals throughout the CNS with slightly stronger intensities in the hippocampal formation (Figures 2C1–C4 and 3C1–C4). Such an expression pattern points to an astroglial localization of this transporter in line with previous findings (28). A capillary localization could also be confirmed for Lat1. However, in contrast to Oatp1c1, Lat1 is not expressed in the choroid plexus. Moreover, at P6 and, to a lesser extent at P12, Lat1 specific hybridization signals were detected in few neuronal populations such as in pyramidal neurons of the hippocampal CA3 region and the cerebellar granule cells indicating that during early stages of postnatal development, Lat1 is also present in distinct neurons (Figures 2D1–D4 and 4D1–D4). For both transporters Oatp1c1 and Lat1, hybridization signal intensities were significantly reduced at P84 indicating an age-dependent decline in the respective transporter expression.

### Distribution pattern of Mct10

Determination of Mct10 mRNA expression in the mouse brain at P6 and P12 revealed overall only very weak signal intensities in specific areas such as layer 6, and subplate neurons of the cerebral cortex as well as granule cells of the dentate gyrus and of the cerebellar cortex (Figures 1E1–E4 and 2E1–E4). At P21, strong signals could be detected throughout white matter regions suggesting that Mct10 is highly enriched in mature oligodendrocytes. In addition, Mct10 expression was clearly visible in the adult brain in neurons of the dentate gyrus and in distinct hypothalamic nuclei such as the dorsomedial nucleus (Figures 2E1–E4). Overall, Mct10 mRNA expression appeared to increase with increasing age of the animals.

## Discussion

Though the importance of TH transporters for proper TH action in the developing and mature CNS has been widely acknowledged, very limited information has been provided so far concerning the localization of TH transporter candidates both in the human as well as mouse CNS. In particular, the generation and analysis of Mct8 deficient mice have raised many questions since these animals lack any overt neurological symptoms, an unexpected finding in light of the pronounced expression of Mct8 in neuronal populations (15). Indeed, an area- as well as cell-type specific analysis of the thyroidal state revealed overall only a mild T3-deprivation such as in striatal cells (14), a normal T3-status in cerebellar neurons (14, 16) and very subtle changes in the cerebral cortex (29). The latter finding has been explained by the presence of Lat2 in cortical neurons that may compensate for the absence of Mct8 in the mouse brain (11). In support for this hypothesis, we could observe a pronounced neuronal expression of Lat2 throughout the cortex with highest signal intensities at P12. Mct8 and Lat2 may also co-operate in mediating TH transport in pyramidal cells of the hippocampus and in neurons of the hypothalamic paraventricular nucleus where transcripts for both transporters could be detected. Possibly, TRH expressing neurons are dependent on Mct8 and Lat2 for a proper negative feedback regulation within the hypothalamus–pituitary–thyroid axis. In this respect, it would be of most interest to study the TH-sensitivity of these neurons in Mct8/Lat2 double deficient animals.

Our analysis, however, also revealed striking differences in the Mct8 and Lat2 distribution pattern with the striatum, thalamus, and brain stem nuclei as the most evident examples. Based on these observations, we assume that besides Lat2, additional proteins must be present in the mouse brain that can facilitate the transmembrane passage of TH in neurons in the absence of Mct8.

One possible candidate is Mct10 that accepts in addition to aromatic amino acids TH as substrate as well (22). Mct10 protein was localized in neurons of the human hypothalamus (12), but its localization in the mouse brain has not been experimentally addressed yet. Here, we could detect Mct10 transcripts in neurons of the hippocampus where Mct10 showed an overlapping expression with Mct8. In contrast, Mct8 and Mct10 exhibited a complementary distribution pattern in the cerebral cortex, which makes a compensatory function of Mct10 in the absence of Mct8 rather unlikely. Moreover, our recent characterization of Mct10/Mct8 deficient animals did not reveal a pronounced TH deficiency in the CNS (30) indicating that at least in the developing murine brain, Mct10 does not significantly contribute to TH transport processes.

Intense Mct10 specific signals were found in white matter regions of the murine CNS at P21 and P84 suggesting that this transporter is highly expressed only in mature oligodendrocytes. It therefore remains to be investigated, which proteins are involved in supplying immature oligodendrocytes with TH particularly since TH represents a critical factor for proper differentiation of these cells (31, 32).

Previous studies of Mct8 ko mice demonstrated a critical role of this transporter in mediating the uptake of T3 via the BBB and/or the BCSFB (14, 16). This physiological function correlates with a strong expression of Mct8 in choroid plexus structures, tanycytes of the third ventricle as well as in capillary endothelial cells as evidenced by immunohistochemical studies (10, 11). Remarkably, Mct8 mRNA expression could only be detected by ISH in larger vessels while choroid plexus structures and tanycytes displayed strong Mct8 mRNA specific signals. The reason for the discrepancy between transcript and protein levels in capillary Mct8 expression cannot be solely related to possible limitations of the ISH procedure since smaller capillaries throughout the brains were successfully visualized with radioactive Oatp1c1 and Lat1 specific RNA probes. It therefore needs to be further assessed in a cell-specific manner how Mct8 expression is regulated on the transcriptional as well as on the translational level.

While in mice, inactivation of Oatp1c1 alone had only mild consequences on the thyroid state of the CNS (27), Mct8/Oatp1c1 dko mice displayed a highly diminished transport of T4 and T3 into the CNS, a finding that underscores the concerted function of Mct8 and Oatp1c1 in facilitating the brain entry of TH via the BBB and/or BCSF (20). Moreover, brain TH concentrations in Mct8/Oatp1c1 were reduced to 10% of the respective wild-type levels indicating a robust hypothyroid state in the CNS. However, it is currently unclear by which pathway the residual TH enters the brain in these animals. In this process, Lat1 may possibly be involved as our ISH analysis revealed a preferential expression of this transporter in capillary cells as well. The exact physiological contribution of Lat1 for TH traffic in the brain will only been unraveled following the generation and analysis of the respective mouse mutants that are either deficient in Lat1 alone or lack even all three TH transporters.

In summary, our ISH analysis revealed a distinct and unique temporal and spatial mRNA expression pattern for all five TH transporter candidates in the murine brain. Although we cannot provide any information about the respective protein levels and subcellular localization, our study will provide a solid ground for determining the cell-specific function of these transporters by taking advantage of conditional mouse mutants. Our data, however, also disclosed that additional TH transporter proteins need to be discovered in order to fully understand TH traffic in such a complex and important TH target organ as the CNS.

## Conflict of Interest Statement

The authors declare that the research was conducted in the absence of any commercial or financial relationships that could be construed as a potential conflict of interest.
